# The role of the Wnt signaling pathway in incretin hormone production and function

**DOI:** 10.3389/fphys.2012.00273

**Published:** 2012-07-12

**Authors:** Yu-ting A. Chiang, Wilfred Ip, Tianru Jin

**Affiliations:** ^1^Department of Physiology, University of TorontoToronto, ON, Canada; ^2^Toronto General Research Institute, University Health NetworkToronto, ON, Canada; ^3^Institute of Medical Science, University of TorontoToronto, ON, Canada

**Keywords:** Wnt signaling pathway, GLP-1, GIP, TCF7L2, insulin, β-catenin

## Abstract

Glucose metabolism is tightly controlled by multiple hormones and neurotransmitters in response to nutritional, environmental, and emotional changes. In addition to insulin and glucagon produced by pancreatic islets, two incretin hormones, namely glucagon-like peptide-1 (GLP-1) and gastric inhibitory polypeptide (GIP, also known as glucose-dependent insulinotropic peptide), also play important roles in blood glucose homeostasis. The incretin hormones mainly exert their regulatory effects via their corresponding receptors, which are expressed in pancreatic islets as well as many other extra-pancreatic organs. Recent studies have shown that the genes which encode these two incretin hormones can be regulated by the effectors of the Wnt signaling pathway, including TCF7L2, a transcription factor identified recently by extensive genome wide association studies as an important type 2 diabetes risk gene. Interestingly, TCF7L2 and β-catenin (β-cat), another effector of Wnt signaling pathway, may also mediate the function of the incretin hormones as well as the expression of their receptors in pancreatic β-cells. In this review, we have introduced the incretin hormones and the Wnt signaling pathway, summarized recent findings in the field, and provided our perspectives.

## Introduction

The metabolic syndrome is a multifactorial disease involving complex interactions between aging, genetics, behavior, and environment. Several peptides, hormones, and neurotransmitters are involved in maintaining glucose and energy homeostasis in response to nutritional, environmental, and emotional changes. In response to fasting and feeding, pancreatic islet α-cells and β-cells secrete glucagon and insulin, respectively, to maintain blood glucose homeostasis. In the beginning of the 19^th^ century, scientists observed that oral glucose administration generates a much stronger insulin secretory response from pancreatic β-cells compared to intravenous injection of equal amounts of glucose (Bayliss and Starling, 1902). Hence, incretins are defined as a group of gastrointestinal hormones that potentiate insulin release from pancreatic islet α-cells after food ingestion, prior to the elevation of blood glucose levels. The first incretin, gastric inhibitory polypeptide (GIP, also known as glucose-dependent insulinotropic peptide) was discovered by Brown and colleagues in the 1970's (Brown, 1971; Brown and Dryburgh, 1971; Dupre et al., 1973; Brown et al., 1975; Ross et al., 1977). About a decade later, the cDNA encoding glucagon in fish, rodents, and humans was isolated which lead to the discovery of the second incretin, glucagon-like peptide-1 (GLP-1) (Bell et al., 1983a,b; Philippe et al., 1986).

GLP-1 is encoded by the proglucagon gene (*gcg*) and is produced in intestinal L cells. The same *gcg* is also expressed in pancreatic α-cells, where it encodes the key glucose-elevating hormone glucagon. As intestinal GLP-1 and pancreatic glucagon are encoded by the same *gcg* gene but exert opposite effects on glucose homeostasis, great efforts have been made to decipher signaling pathways that regulate *gcg* expression in a tissue-specific manner (Lu et al., 1996; Jin et al., 1997; Ni et al., 2003; Jin, 2008). Our group has demonstrated that the two key effectors of the Wnt signaling pathway, β-catenin (β-cat) and TCF7L2, specifically up-regulate *gcg* mRNA expression and GLP-1 production in gut endocrine L cells (Ni et al., 2003; Yi et al., 2005). Shortly after we presented this finding (Ni et al., 2003; Yi et al., 2005), TCF7L2 was identified as an important type 2 diabetes (T2D) risk gene in a large-scale genome-wide association study (GWAS) (Grant et al., 2006). Specifically, two single nucleotide polymorphisms (SNPs) within the intronic regions of TCF7L2 are robustly associated with the risk of T2D. This important finding has been confirmed in numerous studies involving different ethnic groups during the past five years (Grant et al., 2010). Interestingly, it was demonstrated that the Wnt signaling pathway and the TCF effector also regulate the expression of *gip* (Garcia-Martinez et al., 2009). Furthermore, recent investigations revealed that the effectors of the Wnt signaling pathway may modulate the functions of the two incretin hormones as well, and that TCF7L2 regulates the expression of the GLP-1 receptor (GLP-1R) and GIP receptor (GIPR) in pancreatic β-cells (Liu and Habener, 2008; Shu et al., 2009). Finally, the Wnt effectors β-cat and TCF7L2 have been shown to play potential metabolic roles in organs other than the pancreas and gut. In this article, we first provide a brief overview of the incretin hormones and the Wnt signaling pathway. We then summarize recent findings on the role of Wnt signaling in incretin hormone production and function. This is followed by the presentation of our views and perspectives. Mechanistic exploration of the function of the two incretin hormones has been summarized in numerous recent reviews elsewhere (Brubaker and Drucker, 2004; Hansotia and Drucker, 2005; Baggio and Drucker, 2007; Ussher and Drucker, 2012).

## Incretin hormones

In 1902, two English physiologists, Sir William Maddock Bayliss and Ernest Henry Starling speculated that intestinal mucosa contains a hormone which stimulates endocrine secretions from the pancreas after the ingestion of carbohydrates (Bayliss and Starling, 1902). They coined the term “secretin” for the yet to be identified hormone, which even preceded the discovery of insulin in 1921 by the team consisting of Frederick Banting, Charles Best, John Macleod, and James Collip. In the 1930s, the terms “incretin” or “enterogastrone” were proposed by several scientists for a hormonal extract from the duodenum (Cho and Kieffer, 2010). Since the glucose lowering effect was not reproducibly appreciable and experimental methods to reliably measure insulin secretion were lacking at that time, research in this field was not actively pursued until nearly half a century later.

**Figure 1:**
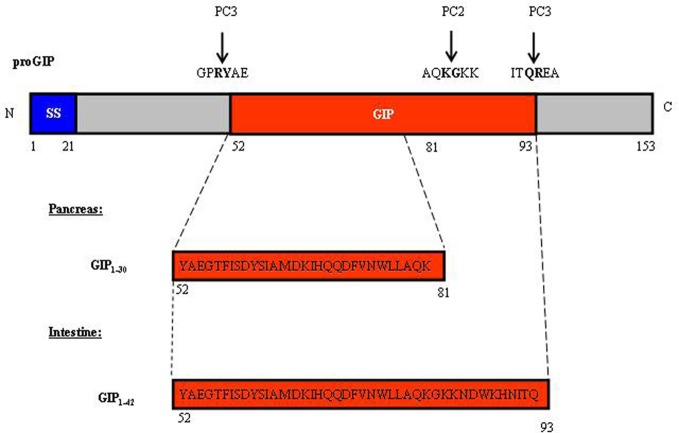
**Structure of proGIP and active GIPs.** The proGIP, encoded by the *gip* gene, is a pro-hormone with 153 amino acid residues. The main circulating active hormone GIP_1–42_ (aa 52–81) is generated predominantly in the intestinal K cells by the pro-hormone convertase PC3. In pancreatic α-cells and in a small portion of intestinal K cells, the cleavage by both PC2 and PC3 leads to the generation of GIP_1–30_ (aa 52–93). SS, signal sequence.

**Figure 2:**
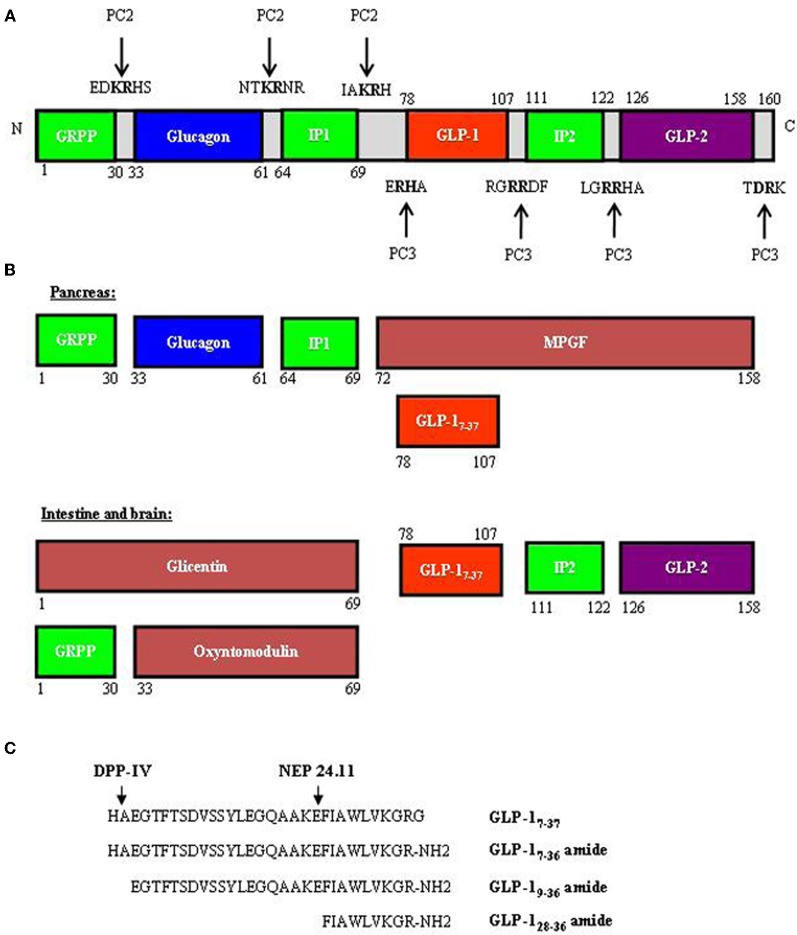
**Proglucagon and its cleavage products (proglucagon derived peptides, PGDPs) including GLP-1. (A)** Proglucagon, encoded by *gcg*, is a pro-hormone with 160 amino acid residues. The peptide contains both PC2 and PC3 cleavage sites. **(B)** A schematic presentation of the cleavage products in the pancreas (top) and in the intestine and brain (bottom). Although GLP-1 is not normally produced in the pancreas, during the embryonic stage or when islets are under stress, some pancreatic α-cell will produce GLP-1. **(C)** Amino acid sequences of GLP-1 and its derivatives, consisting of GLP-1_7–37_ (aa 78–107), GLP-1_7–36_ amide (aa 78–106), GLP-1_9–36_ (aa 80–106), and GLP-1_28–36_ amide (aa 98–106). GRPP, glycentin related polypeptide; IP1 and IP2, intervening peptide 1 and 2; MPGF, major proglucagon fragment; DPP-IV, dipeptidyl peptidase-4; NEP 24.11, neutral endopeptidase 24.11.

The new era of incretin research started in the 1970's with the recognition of the glucose-lowering effect of GIP (Dupre et al., 1973; Brown et al., 1975; Ross et al., 1977), followed by the identification of GLP-1 in the mid-1980's (Bell et al., 1983b; Scott et al., 1983). The majority of GIP is produced by intestinal endocrine K cells in the mucosa of the duodenum and jejunum; however, the *gip* gene has also been shown to be expressed in the pancreatic α-cells (Fujita et al., 2010). As shown in Figure 1, GIP is encoded by the *gip* gene and is derived from a 153 amino acid pro-hormone, proGIP. In the gut, post-translational processing by the prohormone convertase PC1/3 (PC3) leads to the production of the biologically active hormone GIP_1−42_. However, a small population of the K cells also express PC2, leading to the production of lesser amounts of GIP_1−31_ and GIP_1−31_ amide (Cho and Kieffer, 2010). In pancreatic α-cells, proGIP is processed by both PC3 and PC2 to yield GIP_1−31_. GLP-1 is produced by intestinal endocrine L cells throughout the entire small intestine and colon, with highest levels generated within the distal ileum and colon (Hansotia and Drucker, 2005). *Gcg*, the gene that encodes GLP-1 in the gut, also encodes glucagon in pancreatic α-cells. In addition, it encodes GLP-2 in the gut, which functions as a growth factor for the small intestine (Drucker et al., 1996). Figure 2A shows the overall structure of the prohormone and the cleavage sites by PC2 and PC3. In pancreatic α-cells, the main products generated are glucagon, glicentin-related pancreatic polypeptide (GRPP), intervening peptide-1 (IP1) and major proglucagon fragment (MPGF) (Figure 2B). During embryonic stages or after pancreatic islets encounter stress, small amounts of GLP-1 can be detected in pancreatic α-cells (Figure 2B). In the intestine and brain, the post-translational products include glicentin, GLP-1, GLP-2, intervening peptide-2 (IP2), GRPP, and oxyntomodulin (Figure 2B). Figure 2C shows the different GLP-1 derivatives, as well as the cleavage sites of the GLP-1 degrading enzymes dipeptidyl peptidase IV (DPP-IV) and NEP 24.11 (Figure 2C). GLP-1_7−37_ and GLP-1_7−36_ amide are the two biologically active forms. GLP-1_9−36_ amide was previously assumed to be inactive, but was subsequently shown to have protective effects in the heart (Nikolaidis et al., 2004; Zhao et al., 2006; Ban et al., 2009; Noyan-Ashraf et al., 2009; Ban et al., 2010; Cho and Kieffer, 2011). Very recently, the short peptide GLP-1_28−36_ amide was demonstrated to exhibit beneficial metabolic effects in the pancreas and liver (Tomas et al., 2011a,b; Liu et al., 2012).

The recognition of the metabolic effects of the incretin hormones led to the creation of the term “enteroinsular axis”, defined as the connection between the gut and pancreatic islets (Unger and Eisentraut, 1969). Oral nutrient administration (carbohydrate and fat-rich meal) is a potent stimulus of GLP-1 and GIP secretion. Plasma GIP peaks at 15–30 min after oral glucose ingestion (Brown et al., 1975), while a biphasic increase in plasma GLP-1 can be observed in humans, peaking once at 15–20 min and again at 1–2 h following a meal (Elliott et al., 1993; Rask et al., 2001). The half-lives of the native incretin hormones are very short due to the cleavage by DPP-IV, preventing them from being utilized as therapeutic drugs directly. Two new categories of T2D drugs, however, have been developed based on the glucose-lowering effect of GLP-1: GLP-1 analogs such as exenatide (or exendin-4) and liraglutide, and inhibitors of DPP-IV such as sitagliptin and vildagliptin (Wideman and Kieffer, 2009).

Extensive investigations in the past two decades have revealed both overlapping and contrasting actions of the two incretin hormones. Both GLP-1 and GIP exert their functions mainly through their respective receptors, both of which belong to the seven-transmembrane domain G-protein coupled receptor (GPCR) super-family (Brubaker and Drucker, 2004; Hansotia and Drucker, 2005). Cyclic AMP (cAMP) and calcium have been recognized as second messengers for both incretin hormones. Although initial studies focused on the effects of these two hormones in pancreatic β-cells, GLP-1R and GIPR have been located on cells in other organs and the extra-pancreatic effects of these two hormones have been actively investigated.

### Pancreatic functions of GLP-1 and GIP

Pancreatic functions of GLP-1 and GIP principally involve the stimulation of insulin secretion in a synergistic manner with glucose via the closure of ATP-sensitive K^+^ channels (K_ATP_), resulting in subsequent membrane depolarization, rise in intracellular Ca^2+^ level, and Ca^2+^-induced insulin secretion (Light et al., 2002; MacDonald et al., 2002). Both protein kinase A (PKA) and exchange protein activated by cAMP (Epac) signaling pathways were found to be involved in this process (Kwan et al., 2007). GLP-1 and GIP were also shown to stimulate insulin secretion via inhibiting voltage-dependent K^+^ channels (MacDonald et al., 2002). In addition, GLP-1 as well as GIP increased insulin mRNA levels, possibly through stimulating insulin gene transcription and mRNA stability. GLP-1 inhibits glucagon secretion from α-cells and stimulates the secretion of somatostatin from δ-cells (D'Alessio et al., 1989; Komatsu et al., 1989). The stimulation of somatostatin secretion by GLP-1 could be directly mediated by its receptor on pancreatic islet δ-cells, while the inhibition of glucagon secretion could be indirectly mediated through the inhibition of somatostatin and the stimulation of insulin secretion. Additional pancreatic effects of GLP-1 include the sensitization of β-cells to glucose, as well as the induction of proliferation and neogenesis of β-cells (Xu et al., 1999; Tourrel et al., 2001; Li et al., 2003). GLP-1 and exendin-4 (a GLP-1R agonist) were also shown by our group and others to reduce the expression level of thioredoxin-interacting protein (TxNIP), a mediator of glucotoxicity (Chen et al., 2006; Shao et al., 2010). This effect relies on proteasome-mediated TxNIP degradation, involving both PKA and Epac signaling cascades (Shao et al., 2010). Thus, in pancreatic β-cells, the beneficial effects of GLP-1 consist of its metabolic effect on stimulating insulin secretion, its proliferative effect on stimulating β-cell growth and neogenesis, and its protective effect on reducing glucotoxicity (Yu and Jin, 2010). GIP has not been shown to reduce β-cell TxNIP level.

### Extra-pancreatic effects of GLP-1 and GIP

**Figure 3:**
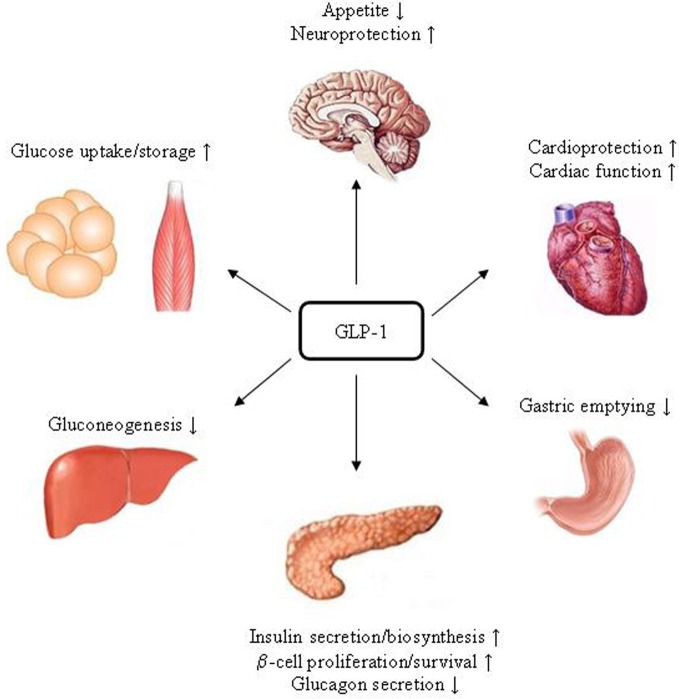
**Schematic presentation of the function of GLP-1.** In the pancreas, stomach, heart and brain, the effects of GLP-1 are likely to be mediated by its specific receptor GLP-1R. As GLP-1_9–37_ was also shown to exert protective effects in the heart and improve cardiac function, whether there is a yet to be identified receptor is under debate.

**Figure 4:**
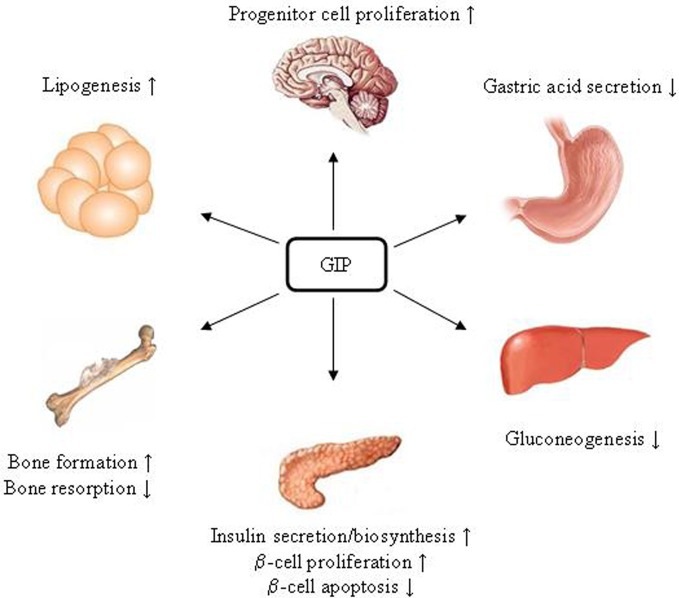
**Schematic presentation of GIP.** Since GIPR has not been detected in hepatic cells, the effect of GIP on reducing gluconeogenesis is likely due to an indirect mechanism.

GLP-1R is expressed in tissues including pancreatic islet β- and δ-cells, lung, stomach, heart, intestine, kidney, and certain brain neurons. Whether it is expressed in hepatocytes is controversial (Bullock et al., 1996; Ding et al., 2006; Gupta et al., 2010; Pedersen and Holst, 2011). Figure 3 summarizes pancreatic and extra-pancreatic functions of GLP-1. It inhibits gastric emptying and attenuates the postprandial rise in plasma glucose (Wettergren et al., 1993; Meier et al., 2003). GLP-1 also exhibits cardio-protective effects in experimental models of cardiac injury (Sokos et al., 2006; Ban et al., 2008, 2010). In the brain, GLP-1 inhibits food intake, presumably via the activation of the GLP-1R in the hypothalamus and brainstem (Turton et al., 1996; Knauf et al., 2005, 2008; Hayes et al., 2008, 2009). Exending-4 exerts the effect in hepatocytes, skeletal muscle, and adipocytes *in vivo*. However, since GLP-1R expression in those tissues is questionable, these effects could be due to indirect mechanisms (Baggio and Drucker, 2007). GIPR is expressed in tissues including the pancreas, stomach, small intestine, adipose tissue, heart, testis, endothelial cells, bone, spleen, thymus, and brain neurons. GIP plays a role in neural progenitor cell proliferation and behavior modification (Nyberg et al., 2005). It also stimulates lipogenesis and bone formation (Zhong et al., 2007). Figure 4 summarizes the effects of GIP. Detailed descriptions of function of these two incretin hormones have been summarized in many excellent review articles (Brubaker and Drucker, 2004; Estall and Drucker, 2006; Ussher and Drucker, 2012).

## Wnt signaling pathway and TCF7L2

The importance of the Wnt signaling pathway was initially recognized in colon cancer studies and research on embryonic development in *Drosophila, Xenopus*, and other organisms. Wnt ligands, through their cell membrane bound receptors and co-receptors, exert many fundamental physiological and pathophysiological functions in multiple organs and cell lineages, including organogenesis, tumorigenesis, and the recently-identified functions in metabolic homeostasis.

**Figure 5:**
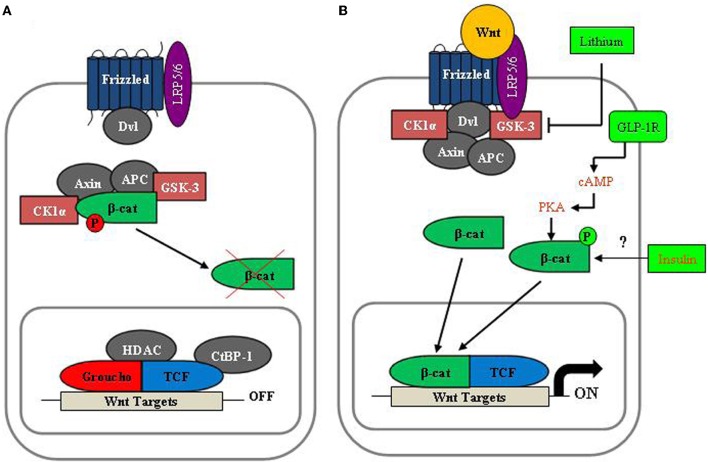
**A simplified illustration of the canonical Wnt signaling pathway. (A)** In the absence of Wnt ligand stimulation, β-cat is located within the “destruction complex”, phosphorylated by GSK-3 and CK-1α at Ser33 and adjacent serine positions, which leads to proteasome-mediated degradation process. **(B)** Following Wnt ligand stimulation, the destruction complex disassembles, resulting in free β-cat accumulation. It then enters the nucleus and forms the bipartite transcription factor β-cat/TCF, which leads to the stimulation of Wnt target gene expression. Lithium is an inhibitor of GSK-3, while cAMP signaling and insulin are able to stimulate β-cat Ser675 phosphorylation. Thus, in certain cell lineages, β-cat/TCF can also mediate the effect of lithium, cAMP, and insulin on Wnt target gene expression.

As shown in Figure 5A, the key downstream effector of the canonical Wnt signaling pathway (defined as Wnt pathway hereafter) is the bipartite transcription factor β-cat/TCF, formed by β-cat and a member of the TCF family (TCF7, LEF-1, TCF7L1, and TCF7L2). The level of cytosolic β-cat is tightly controlled by the proteasome-mediated degradation process, involving the tumor suppressor adenomatous polyposis coli (APC), axin/conductin, as well as the serine/threonine kinases glycogen synthase kinase-3 (GSK-3) and casein kinase 1α (CK1α). β-cat is phosphorylated by GSK-3 and CK1α at Ser33 and several other adjacent serine and threonine positions to facilitate its degradation. Wnt ligands exert their regulatory effects via the seven-transmembrane domain frizzled receptors and the low-density lipoprotein receptor-related proteins 5 and 6 (LRP5/6) co-receptors. Following Wnt ligand binding, the Wnt receptor associates with disheveled (Dvl) (Figure 5B). This event triggers the disruption of the complex containing APC, axin, GSK-3, and β-cat, thus preventing the phosphorylation-dependent degradation of β-cat. In turn, β-cat enters the nucleus to form the β-cat/TCF complex, resulting in the activation of β-cat/TCF (or Wnt) downstream target genes (Figure 5B). GSK-3 is an important negative modulator of the Wnt signaling pathway. Lithium and other inhibitors of GSK-3 have been shown to mimic the function of Wnt ligands in stimulating the expression of Wnt downstream target genes (Figure 5B). It should be pointed out that extensive recent studies have revealed that β-cat/TCF also mediates the effect of signaling cascades other than the Wnt signaling pathway (Jin et al., 2008). A battery of peptide hormones that utilize cAMP as their second messenger, insulin, insulin-like growth factor-1 (IGF-1) and other growth factors, as well as the lipid metabolite lysophosphatidic acid (LPA) have been shown to exert their effects through the Wnt effector β-cat/TCF (Jin et al., 2008). Notably, PKA activation leads to β-cat Ser675 phosphorylation, an event that is positively associated with increased β-cat/TCF transcriptional activity (Hino et al., 2005; Taurin et al., 2006). We and others have shown that β-cat Ser675 phosphorylation can be stimulated by insulin and IGF-1 in *gcg*-expressing gut endocrine L cells and in other cell lineages (Sun et al., 2009, 2010), while a very recent study showed that β-cat can be phosphorylated by p21-activated protein kinase 1 (Pak1) (Zhu et al., 2012).

Although TCF7 (TCF-1) was originally isolated as a lymphoid transcription factor, members of this family are now well recognized to be transcriptional regulators of many physiological processes. Shortly after the identification of TCF-1/TCF7, Castrop et al. isolated cDNAs for TCF7L1 and TCF7L2, which they named TCF-3 and TCF-4, respectively, (Castrop et al., 1992). Because the high-mobility group (HMG) boxes of the TCF7L1, TCF7L2, and TCF7 sequences show striking similarity, Castrop et al. suggested that they represent a subfamily of HMG box-containing transcription factors (Castrop et al., 1992). Human TCF7L2 gene was mapped to chromosome 10q25.3 (Duval et al., 2000).

**Figure 6:**
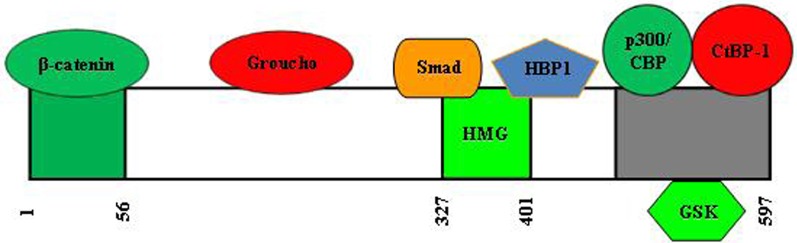
**Structure and functional domains of human TCF7L2.** It is currently unclear which position of the domains that interact with Groucho and CtBP1. Smad4 is the major mediator of the TGFβ signaling cascade. HBP1, HMG-box transcription factor 1, a transcriptional repressor.

**Figure 7:**
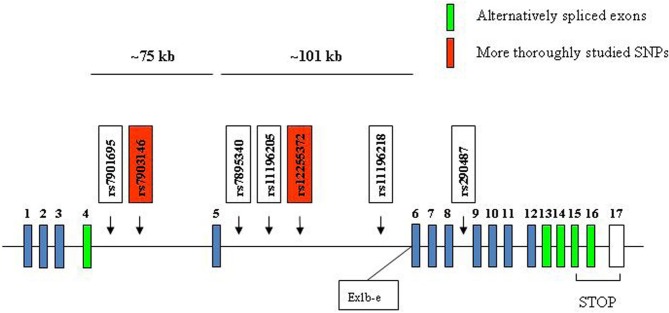
**Depiction of the position of seven T2D risk SNPs in the human TCF7L2 gene.** Human TCF7L2 is located on chromosome 10q25.3. The two SNPs that have been more extensively studied are shown in red. Ex1b-e indicates the relative position of a novel promoter, which is active in neuronal cells during embryonic stages and leads to the generation of dominant negative TCF7L2, lacking the first 161 amino acid residues at the N terminus.

Figure 6 shows the domain structure of TCF7L2. In the absence of Wnt ligand stimulation, TCF7L2 or other HMG box TCF proteins function in the nucleus as transcriptional repressors of Wnt target genes. TCF forms a complex with transcriptional co-repressors, including Groucho and C-terminal binding protein 1 (CtBP-1). Both Groucho and CtBP-1 are able to recruit nuclear co-repressors, such as histone deacetylases (HDACs), to the promoters of Wnt target genes. β-cat, however, is able to convert TCF into a transcriptional activator for the same panel of genes that are repressed by TCF in the absence of β-cat. The first 41 amino acids of TCF7L2 form the β-cat binding domain. Deletion of this domain generates a dominant negative molecule (Yi et al., 2005). A dominant negative form of TCF7L2 was shown to inhibit the ability of constitutively-active β-cat to stimulate TCF-dependent transcription (Kolligs et al., 1999). This dominant negative molecule was shown by our group to block both basal and lithium-stimulated *gcg* expression in a mouse intestinal endocrine L cell line (Yi et al., 2005). Very recently, Vacik et al. demonstrated in the mouse brain the existence of a novel TCF7L2 promoter located upstream of exon 6 (Figure 7). Transcription from this alternative promoter leads to the generation of a 35 kDa TCF7L2 protein, lacking the first 161 amino acid N-terminal portion, which functions as a native dominant-negative TCF7L2 protein in the brain (Vacik et al., 2011). Whether such a dominant negative molecule is also expressed in certain peripheral tissues is currently unknown.

In 1999, Duggirala and colleagues reported that a region on chromosome 10q was linked to T2D in Mexican Americans (Duggirala et al., 1999). Reynisdottir et al. had also obtained evidence for a potential linkage of T2D to 10q in an Icelandic population (Reynisdottir et al., 2003). In 2006, Grant et al. reported their discovery of the potential linkage between polymorphisms in TCF7L2 and the risk of T2D (Grant et al., 2006). They genotyped 228 microsatellite markers in Icelandic individuals with T2D and healthy controls across a 10.5 Mb interval on chromosome 10q. Microsatellite, DG10S478, located within intron 4 of the TCF7L2 gene, was found to be associated with the risk of T2D. This observation was then replicated in a Danish cohort as well as a U.S. cohort (Grant et al., 2006). Two SNPs, namely rs12255372 and rs7903146, were found to be in strong linkage disequilibrium with DG10S478 and also showed similar robust associations with T2D. Heterozygous and homozygous carriers of the at-risk alleles had relative T2D risks of 1.45 and 2.41, respectively, (Grant et al., 2006). During the past six years, the findings in this hallmark publication have been repeated in many ethnic groups by numerous research teams (Cauchi et al., 2006; Florez et al., 2006; Groves et al., 2006; Chang et al., 2007; Duan et al., 2007; Florez, 2007, 2008; Lyssenko et al., 2007, 2008; Schafer et al., 2007; Gonzalez-Sanchez et al., 2008; Ng et al., 2008; Shu et al., 2008, 2009; Cornelis et al., 2009; da Silva Xavier et al., 2009; Gloyn et al., 2009; Pilgaard et al., 2009; Alibegovic et al., 2010; Groop, 2010; Dabelea et al., 2011; Gjesing et al., 2011). As shown in Figure 7, the T2D risk TCF7L2 SNPs are mainly localized within the two large intronic regions surrounding exon 5. Although it will be challenging to determine how these SNPs affect the risk of T2D, several studies suggest that these SNPs affect the incretin effect of pancreatic β-cells and hepatic gluconeogenesis (Lyssenko et al., 2007, 2008; Shu et al., 2008, 2009). Since there are a number of alternatively spliced TCF7L2 isoforms, it has been suggested that the risk SNPs affect the expression as well as the alternative splicing of TCF7L2.

## Wnt pathway controls incretin gene expression

We have demonstrated previously that both lithium (which mimics the function of Wnt ligands) and a constitutively-active β-cat (S33Y mutant) stimulated the activity of the *gcg* promoter (Ni et al., 2003). Lithium was also shown to stimulate endogenous *gcg* mRNA expression and GLP-1 production in the mouse intestinal GLUTag and STC-1 cell lines, as well as in fetal rat intestinal cell (FRIC) cultures (Ni et al., 2003). We then identified that the stimulatory effect of lithium on *gcg* expression occurred in intestinal endocrine L cells, but not in pancreatic α-cells. Activation of *gcg* promoter activity is dependent upon a TCF binding site within the G2 enhancer element of the *gcg* promoter (Yi et al., 2005). Since the G2 enhancer element has been shown by Furstenau et al. to mediate the stimulatory effects of both cAMP and calcium on *gcg* promoter activity (Furstenau et al., 1999), this observation raised the question as to whether cAMP activates *gcg* expression via cross-talking with the Wnt signaling pathway. Using chromatin immunoprecipitation (ChIP), we then demonstrated an *in vivo* physical interaction between TCF7L2 and the G2 enhancer element (Yi et al., 2005). Western blotting, RT-PCR, and immunostaining demonstrated that TCF7L2 is abundantly expressed in intestinal GLP-1 producing cells (Yi et al., 2005). Furthermore, dominant-negative TCF7L2 attenuated both basal and lithium-stimulated *gcg* mRNA expression in the intestinal endocrine L cell line GLUTag (Yi et al., 2005). Interestingly, *gip* mRNA expression was also shown to be stimulated by the Wnt signaling cascade (Garcia-Martinez et al., 2009). García-Martínez and colleagues demonstrated that lithium or Wnt/β-cat signaling enhances GIP production by entero-endocrine cells through a conserved TCF binding site within the proximal region of the *gip* promoter. In contrast to the positive effect of TCF7L2 on *gcg* promoter transcription, ChIP analysis showed that lithium treatment led to a favorable binding of the bipartite transcription factor of β-cat/LEF-1 to the *gip* promoter, reducing the binding of TCF7L2 and the nuclear co-repressor histone deacetylase 1 (HDAC1) (Garcia-Martinez et al., 2009). Thus, although Wnt signaling positively regulates the expression of both GIP and GLP-1, they are differentially regulated by the effectors LEF-1 and TCF7L2, respectively, (Garcia-Martinez et al., 2009).

## Potential effect of Wnt effectors β-cat and TCF7L2 in pancreatic β-cells

To explore the mechanistic role of TCF7L2 SNPs in conferring the risk of T2D, Schafer *et al* genotyped 1100 non-diabetic German participants for the five known TCF7L2 SNPs and conducted oral glucose tolerance tests on these subjects (Schafer et al., 2007). They then measured GLP-1 secretion and performed intravenous glucose tolerance tests in a portion of the participants. Their results confirmed that TCF7L2 SNPs are associated with reduced insulin secretion. Plasma GLP-1 concentrations during oral glucose tolerance tests, however, were not correlated with the status of TCF7L2 SNPs. These observations indicate that TCF7L2 polymorphisms may mainly affect the incretin response rather than the production of the incretin hormone. Many investigators have thus focused on assessing the function of TCF7L2 in pancreatic β-cells (Shu et al., 2008, 2009; Liu and Habener, 2008).

A series of studies by Maedler's group revealed the beneficial effects of TCF7L2 in pancreatic β-cells (Shu et al., 2008, 2009). They found that in isolated human islets, siRNA-mediated TCF7L2 depletion resulted in increased β-cell apoptosis, decreased β-cell proliferation, and decreased glucose-stimulated insulin secretion. Similar effects were seen following TCF7L2 depletion in mouse islets. In contrast, TCF7L2 over-expression protected islets from glucose- and cytokine-mediated apoptosis of pancreatic β-cells. They concluded that TCF7L2 plays a beneficial role on both β-cell function and survival. They then investigated the correlation between the pancreatic level of TCF7L2 and the levels of GLP-1R and GIPR. In the diabetic *db*/*db* mouse model, the Vancouver Diabetic Fatty (VDF) Zucker rat and the high fat/high sucrose diet-treated mouse model, TCF7L2 protein levels were lower in the diabetic animals despite an increase in TCF7L2 mRNA levels in isolated islets compared to control animal islets. A similar trend was also observed in pancreatic sections from patients with T2D. In parallel, expression of GLP-1R and GIPR was also lower in islets from humans with T2D as well as in isolated human islets treated with TCF7L2 siRNA. Also, glucose-, GLP-1-, and GIP-stimulated insulin secretion, but not KCl- or cAMP-stimulated insulin secretion, was impaired in TCF7L2 siRNA-treated isolated human islets. Loss of TCF7L2 resulted in decreased GLP-1- and GIP-stimulated AKT phosphorylation, and AKT-mediated Foxo-1 phosphorylation and nuclear exclusion. These findings suggest that β-cell function and survival are positively regulated through the interplay between TCF7L2 and GLP-1R/GIPR expression and signaling in T2D. These observations, however, are in contrast with the potential deleterious effect of TCF7L2 in pancreatic β-cells. For example, Lyssenko et al. reported that the CT/TT genotypes of SNP rs7903146 are strongly associated with the risk of T2D in two independent cohorts. This risk T allele was associated with impairments in insulin secretion, incretin effects, and enhanced rate of hepatic glucose production (Lyssenko et al., 2007). Furthermore, the carriers of the TT alleles exhibited increased pancreatic TCF7L2 mRNA level. Although TCF7L2 expression was positively correlated with insulin gene expression, it correlated inversely with glucose-stimulated insulin release (Lyssenko et al., 2007). Based on these observations, we would speculate that the TCF7L2 risk allele is associated with increased TCF7L2 expression, and this is deleterious to the incretin effect and glucose disposal. More recently a transgenic mouse study has also demonstrated the deleterious effect of TCF7L2 in pancreatic islets (Savic et al., 2011). Briefly, TCF7L2 null mice display enhanced glucose tolerance coupled with significantly lowered insulin levels, while TCF7L2 over-expression displayed reciprocal phenotypes (Savic et al., 2011). Great efforts have been made to determine the alternative splicing of TCF7L2 (Osmark et al., 2009; Prokunina-Olsson et al., 2009; Prokunina-Olsson and Hall, 2010; Locke et al., 2011; Savic et al., 2011). It is unclear but possible that different isoforms of TCF7L2 may exert different or even opposite functions (Le Bacquer et al., 2011), which may explain the seemingly controversial conclusions made by different groups on the beneficial versus deleterious role of TCF7L2 in β-cells, as discussed above (Lyssenko et al., 2007; Shu et al., 2008, 2009; da Silva Xavier et al., 2009; Savic et al., 2011).

Liu and Habener assessed the involvement of TCF7L2 and β-cat in glucose disposal from another angle (Liu and Habener, 2008). They assessed the expression of TCF7L2 in the rat pancreatic β-cell line Ins-1 and determined the presence of Wnt activity in pancreatic islets using the TOPGAL transgenic mice. In this mouse model, the expression of the β-galactosidase (LacZ) reporter is under the control of a regulatory sequence consisting of three consensus LEF/TCF binding sites upstream of a minimal c-*fos* gene promoter (DasGupta and Fuchs, 1999). Liu and Habener found that islets from TOPGAL mice show increased LacZ expression in response to exendin-4 treatment, although basal LacZ expression in islets was shown to be low (Liu and Habener, 2008). This observation suggests that β-cat/TCF, the effector of the Wnt signaling, mediates the effect of GLP-1 in stimulating β-cell proliferation. The positive pancreatic LacZ staining in the TOPGAL mice, however, could not be repeated by other investigators (Columbus et al., 2010; Krutzfeldt and Stoffel, 2010).

## Metabolic function of TCF7L2 in organs other than the pancreas and gut

TCF7L2 is also expressed in organs other than pancreatic islets, such as liver, brain, muscle, and fat tissues, which are also important for metabolic homeostasis. Although the Wnt signaling pathway is known to be important in the development and zonation of the embryonic liver (Gebhardt and Hovhannisyan, 2010), little effort has been made to explore the hepatic role of TCF7L2 and Wnt signaling in regulating glucose homeostasis in adulthood. Liu et al. reported that starvation induced the expression of mRNAs encoding different Wnt isoforms in hepatocytes. They also demonstrated with gain- and loss-of-function models that β-cat mediates hepatic glucose production (Liu et al., 2011). Briefly, β-cat ablation improved glucose disposal and inhibited the expression of gluconeogenic gene expression, while β-cat over-expression showed the reciprocal effect (Liu et al., 2011). This group, however, did not directly assess the contribution of TCF7L2. Norton et al. demonstrated that TCF7L2 silencing led to increased basal levels of hepatic glucose production in a rat hepatic cell line, associated with the over-expression of gluconeogenic genes including those encodes phosphoenolpyruvate carboxykinase (*pepck*) and glucose-6-phosphatase (*g6pase*) (Norton et al., 2011). Using ChIP combined with massive parallel DNA sequencing (ChIP-Seq), they detected more than 2000 binding events across the genome (Norton et al., 2011). They suggested that TCF7L2 may affect fasting and postprandial hyperglycemia in carriers of T2D risk SNPs of TCF7L2 (Norton et al., 2011).

Wnt signaling and TCF7L2 negatively regulate adipogenesis (Ross et al., 2000) but positively regulate bone formation (Manolagas and Almeida, 2007). Wnt ligands released by adipocytes stimulate insulin secretion (Schinner et al., 2008). TCF7L2 is expressed in adipocytes and its expression can be down-regulated by insulin (Ahlzen et al., 2008). In cultured adipocytes, insulin repressed TCF7L2 expression, and the repression can be attenuated by free fatty acids palmitate or oleate (Ahlzen et al., 2008). In insulin-resistant human subjects, subcutaneous adipose tissue (SAT) expresses higher levels of TCF7L2 (Ahlzen et al., 2008). Prokunina-Olsson and colleagues found that omental and SAT express different alternatively spliced forms of TCF7L2. However, there is no association between the expression of alternatively spliced TCF7L2 isoforms and TCF7L2 T2D risk SNPs (Prokunina-Olsson et al., 2009).

## Summary and perspective

A fundamentally important advancement in the basic research of the Wnt signaling pathway is that the bipartite transcription factor β-cat/TCF mediates not only the effect of Wnt signaling, but also other signaling pathways, including peptide hormones that utilize cAMP as a second messenger (Jin et al., 2008). This, along with the breakthrough discovery that TCF7L2 is a T2D risk gene through GWAS (Grant et al., 2006), facilitated the investigation of the role of Wnt signaling pathway in metabolic homeostasis. Investigations made during the past few years have shown that the production of GLP-1 and GIP is positively regulated by the effectors of the Wnt signaling pathway (Yi et al., 2005; Garcia-Martinez et al., 2009). Furthermore, β-cat/TCF7L2 mediates the function of GLP-1 in stimulating β-cell proliferation (Liu and Habener, 2008), and that TCF7L2 plays a role in up-regulating the expression of GLP-1R and GIPR (Shu et al., 2009).

Although it is clear that TCF7L2 polymorphisms are strongly associated with the risk of T2D in multiple ethnic groups, the mechanism underlying this association is far from complete at this time. Since no risk SNPs of T2D have been identified within TCF7L2 coding region, or a region that can be reliably determined to have a strong effect on TCF7L2 expression or alternative splicing, we still cannot rule out the possibility that the risk SNPs of TCF7L2 affect T2D susceptibility via a mechanism that does not involve TCF7L2 itself, but rather that the SNPs are evolutionarily linked to the inheritance of a genetic defect elsewhere (Jin and Liu, 2008). In the future, we anticipate more thorough investigation on the association between TCF7L2 SNPs and the alternative splicing of TCF7L2 in pancreatic islets and other organs. Great insights could also be gained through further studies on the beneficial versus deleterious effects of TCF7L2 alternatively spliced variants in different organs.

Both TCF7L2 expression and Wnt activity have been clearly demonstrated in adult organs including liver, adipocytes, and brain. These organs are important in regulating glucose and energy homeostasis, as well as eating behavior. In the liver, TCF7L2 is likely to act as negative mediators of gluconeogenesis (Norton et al., 2011). However, β-cat ablation has been shown to improve glucose disposal and inhibit gluconeogenic gene expression (Liu et al., 2011). This is likely due to the fact that the FOXO signaling pathway is also importantly involved in hepatic gluconeogenesis and that FOXO cross-talks with the Wnt signaling pathway (Manolagas and Almeida, 2007; Jin et al., 2008; Norton et al., 2011). An evolutionarily conserved interaction between β-cat and FOXO proteins was demonstrated by Essers et al. in 2005 (Essers et al., 2005). We hence learned that FOXO and TCF compete for their common co-factor β-cat in order to exert their physiological or pathophysiological functions (Jin et al., 2008). FOXO mediates the effect of glucagon signaling in stimulating the transcription of gluconeogenic genes including *pepck* during hypoglycemia, which is turned off by insulin during hyperglycemia by insulin. The attenuation of the expression of *pepck* and other gluconeogenic genes by knocking down β-cat suggests that FOXO requires β-cat to stimulate *pepck* transcription (Liu et al., 2011). How does one explain the contrasting observation that both Wnt signaling and another effector of Wnt signaling pathway, TCF7L2, negatively regulate gluconeogenic gene expression (Norton et al., 2011)? One may speculate that β-cat is a limiting factor for the FOXO signaling cascade in up-regulating gluconeogenic gene expression during fasting, while the repression of gluconeogenic gene expression by Wnt activation or TCF7L2 over-expression does not rely on β-cat as a limiting factor after feeding. Alternatively, TCF7L2 may utilize a yet to be explored mechanism or co-factor to repress gluconeogenic gene expression. These potential mechanisms deserve further investigation.

Adipocytes also express TCF7L2. In human adipocytes, TCF7L2 expression can be down-regulated by insulin (Ahlzen et al., 2008), in contrast to the stimulatory effect of insulin on TCF7L2 expression in pancreas and gut (Columbus et al., 2010; Sun et al., 2010). Insulin is a potent inducer of adipogenesis, while Wnt signaling is known to inhibit adipogenesis (Ross et al., 2000; Ahlzen et al., 2008; Schinner, 2009). Down-regulation of TCF7L2 by insulin in adipocytes may hence mediate the stimulation of adipogenesis by insulin. This raises the question as to whether known adipogenic genes are negatively regulated by TCF7L2. If they are, what is the co-factor for TCF7L2 in mediating its repressive effect? This line of research will broaden our knowledge on adipogenesis and provide potential novel targets for controlling obesity. Insulin is known to repress pancreatic *gcg* expression (Philippe, 1991), in contrast to its stimulatory effect on *gcg* expression in the gut (Yi et al., 2008). Since insulin has been shown to exert opposite effects on the expression of TCF7L2 in different tissues, to explore mechanistically how insulin represses TCF7L2 in adipocytes will also broaden our basic knowledge on the role of insulin in regulating gene expression.

Both GLP-1 and GIP are expressed in the brain. We have learned that brain GLP-1 signaling controls satiety (Turton et al., 1996; Baggio and Drucker, 2007), while exogenous GIP administration in the rat induces proliferation of hippocampal progenitor cells (Nyberg et al., 2005). Wnt signaling pathway is evidently involved in neuron cell differentiation and survival. TCF7L2 is expressed in different brain neuronal cell types, and TCF7L2 knockout mice carry abnormalities in their pituitary gland (Brinkmeier et al., 2007). Since TCF7L2 or Wnt signaling pathway play an important role in the expression of peripheral GLP-1 and GIP (Yi et al., 2005, 2008; Garcia-Martinez et al., 2009), it is necessary to examine whether TCF7L2 and Wnt signaling control brain incretin hormone production and function.

In summary, the studies on the expression and function of incretin hormones has enriched our understanding of the mechanisms underlying glucose and energy homeostasis, and have led to the development of two novel categories of therapeutic drugs for T2D and its complications. The recognition of the involvement of Wnt signaling in metabolic homeostasis and the association between TCF7L2 polymorphisms and the risk of T2D further prompted us to explore the role of Wnt signaling and TCF7L2 in the production and function of the incretin hormones. Although majority of the studies have been conducted in pancreatic β-cells, we will likely see more extensive investigations on the role of Wnt signaling and its effectors in organs other than pancreatic islets and potentially the identification of novel therapeutic targets for T2D and other metabolic disorders.

### Conflict of interest statement

The authors declare that the research was conducted in the absence of any commercial or financial relationships that could be construed as a potential conflict of interest.
